# Network pharmacology-based strategy to investigate the mechanisms of artemisinin in treating primary Sjögren’s syndrome

**DOI:** 10.1186/s12865-024-00605-3

**Published:** 2024-02-12

**Authors:** Jia-he Liao, Qian He, Zi-wei Huang, Xin-bo Yu, Jian-ying Yang, Yan Zhang, Wei-jiang Song, Jing Luo, Qing-wen Tao

**Affiliations:** 1 Graduate School, Beijing University of Chinese Medicine, Beijing, China; 2 Traditional Chinese Medicine Department of Rheumatism, China-Japan Friendship Hospital, Beijing, China; 3 Traditional Chinese Medicine Department, Peking University Third Hospital, Beijing, China; 4 Beijing Key Laboratory of Immune Inflammatory Disease, China-Japan Friendship Hospital, Beijing, China

**Keywords:** Artemisinin, Sjögren’s syndrome, Network pharmacology, Regulatory T cells, Th17 cells

## Abstract

**Objective:**

The study aimed to explore the mechanism of artemisinin in treating primary Sjögren’s syndrome (pSS) based on network pharmacology and experimental validation.

**Methods:**

Relevant targets of the artemisinin and pSS-related targets were integrated by public databases online. An artemisinin-pSS network was constructed by Cytoscape. The genes of artemisinin regulating pSS were imported into STRING database to construct a protein-protein interaction (PPI) network in order to predict the key targets. The enrichment analyses were performed to predict the crucial mechanism and pathway of artemisinin against pSS. The active component of artemisinin underwent molecular docking with the key proteins. Artemisinin was administered intragastrically to SS-like NOD/Ltj mice to validate the efficacy and critical mechanisms.

**Results:**

Network Pharmacology analysis revealed that artemisinin corresponded to 412 targets, and pSS related to 1495 genes. There were 40 intersection genes between artemisinin and pSS. KEGG indicated that therapeutic effects of artemisinin on pSS involves IL-17 signaling pathway, HIF-1 signaling pathway, apoptosis signaling pathway, Th17 cell differentiation, PI3K-Akt signaling pathway, and MAPK signaling pathway. Molecular docking results further showed that the artemisinin molecule had higher binding energy by combining with the key nodes in IL-17 signaling pathway. In vivo experiments suggested artemisinin can restored salivary gland secretory function and improve the level of glandular damage of NOD/Ltj mice. It contributed to the increase of regulatory T cells (Tregs) and the downregulated secretion of IL-17 in NOD/Ltj model.

**Conclusion:**

The treatment of pSS with artemisinin is closely related to modulating the balance of Tregs and Th17 cells via T cell differentiation.

**Supplementary Information:**

The online version contains supplementary material available at 10.1186/s12865-024-00605-3.

## Introduction

Primary Sjögren syndrome (pSS) is a common chronic autoimmune disease characterized by highly lymphocytic infiltration of exocrine glands, especially salivary glands and lacrimal glands, which often results in dry eyes, dry mouth, and fatigue [1]. Moreover, it can cause damage to extra-glandular organs and systems such as interstitial pneumonia, interstitial nephritis, and severe thrombocytopenia [2–4]. As a connective tissue disease with a high prevalence of 0.03–2.7% worldwide [5], the pathogenesis of pSS has not been fully elucidated, and is currently considered to be mainly related to genetics, immunodeficiency, viral infections, and environmental factors [6]. B cells, T follicular helper cells, regulatory T cells (Tregs), T helper 17 (Th17) cells, follicular dendritic cells, innate cells, and several cytokines, chemokines, miRNAs such as Interleukin (IL)-17, IL-6, CXCL10, CXCL13, miR-146a have been proved to participate in the development of pSS [7].

At present, the therapeutic agents that are commonly used to treat pSS are mostly empirical, or draw on the treatment of similar lesions [8]. Artificial tears, lubricants, and saliva substitutes are often used to alleviate dryness in pSS. Non-steroidal anti-inflammatory drugs (NSAIDs), analgesics, and corticosteroids are often used for the management of pain and fatigue of pSS [9]. Hydroxychloroquine (HCQ) and methotrexate (MTX) can be used for the treatment of arthralgia, mild to moderate arthritis and skin involvement in pSS [10]. In addition, a number of other disease modifying antirheumatic drugs (DMARDs) have specific manifestations for the treatment of this disease, but are often accompanied by gastrointestinal, hematologic, and other adverse effects [11]. There are still no satisfactory treatment and effective drugs proven by high quality evidence-based medicine for pSS [12]. The early experience in pSS therapeutic trials in repurposing therapies used in other autoimmune rheumatic diseases has often failed to demonstrate efficacy [13]. Thus, there is an urgent need to elucidate the pathogenesis of pSS to develop new effective and safe therapeutic strategies.

Artemisinin is an effective ingredient extracted from the Chinese herbal medicine *Artemisia annua*. It is currently the most effective and low-toxicity antimalarial drug [14]. Artemisinin and its derivatives have been validated for the treatment of different types of tumors, inflammatory and immunomodulatory related diseases, such as rheumatoid arthritis (RA), systemic lupus erythematosus (SLE), multiple sclerosis and allergic diseases, benefiting from its anticancer, anti-inflammatory and anti-infective properties, in addition to antimalarial properties [15, 16]. Experimental validation has shown that artemisinin and its derivatives could exert anti-inflammatory and immunomodulatory properties by regulating T cell subsets and their corresponding functional changes, suppressing activated T cells, and diminishing B cells [17–20]. Therefore, artemisinin has potential to treat pSS, but there is a lack of relevant studies.

Network pharmacology is a widely used technique based on systems biology and biological network equilibrium for identifying the major active molecular compounds of drugs and screening their potential therapeutic targets [21]. Molecular docking is an approach to simulate and calculate the affinity of compounds [22]. This study is aimed to further reveal the mechanism of artemisinin in the treatment of pSS. A network pharmacology approach was used to predict the key targets and pathways of artemisinin; the interactions between key compounds and core proteins of key pathways were verified by molecular docking; the predicted results of network pharmacology were validated by in vivo experiments.

## Materials and methods

### Collecting related targets of artemisinin and pSS

The present study utilized the TCMSP (http://lsp.nwu.edu.cn/tcmsp.php), HERB (http://herb.ac.cn), and ChEMBL (https://www.ebi.ac.uk/chembl) databases to identify potential targets of artemisinin. Pertinent properties of artemisinin, such as molecular weight, oral bioavailability, and drug-likeness, were extracted from the TCMSP database. The target predictions module of the ChEMBL database was employed to screen compound-related targets, with the species set as “*Homo sapiens*” and an active confidence interval of 90%. The HERB database was utilized to conduct a search for the modules “Related Gene Targets” and “Differentially expressed genes” using artemisinin as the keyword. The identified targets were then screened for duplicates as potential targets of artemisinin. To identify pSS-related targets, the OMIM (https://www.omim.org/), GeneCards (https://www.genecards.org/), and PharmGKB (https://www.pharmgkb.org/) databases were employed with “primary Sjögren’s syndrome” as the keyword. The artemisinin-pSS network was constructed using Cytoscape (version 3.9.0).

### PPI network construction

The common targets between artemisinin and pSS were analyzed by a protein-protein interaction (PPI) network using the STRING database (https://string-db.org/,version 11.5). Interaction scores greater than 0.4 were considered statistically significant.

### Enrichment analysis

The study employed Gene Ontology (GO) analysis to ascertain the biological process (BP), cellular component (CC), and molecular function (MF). The Database for Annotation, Visualization, and Integrated Discovery (DAVID, https://david.ncifcrf.gov/, version 6.8) was utilized to conduct GO enrichment analysis of the target proteins of artemisinin that act on pSS. A bar chart was generated to display the filtered biological processes. Additionally, Kyoto Encyclopedia of Genes and Genomes (KEGG) pathway enrichment analysis was conducted to categorize the relevant pathways of the target proteins of artemisinin that act on pSS. Pathways that met the criterion of p<0.05 were deemed statistically significant and subsequently utilized for follow-up experimental validation in relation to pSS.

### Molecular docking

In this study, the core proteins of significant pathways were subjected to molecular docking. The crystal structures of the target proteins were obtained from the RCSB PDB database (https://www.rcsb.org/) and saved as pdbqt files. The structure of artemisinin was acquired from the PubChem database (https://pubchem.ncbi.nlm.nih.gov/) and converted into MOL2 format using Chem3D. The AutoDock Tools (version 1.5.6) software was utilized to perform molecular docking between the protein and compound structures, with atom type specification, binding site identification, and binding energy calculation. All relevant information was subsequently exported as pdbqt files. The molecular docking calculation was executed utilizing the Lamarckian genetic algorithm. The complexes exhibiting higher binding energy were selected for comparison and visualization through PyMOL (version 2.1) to derive the binding patterns of the compounds and proteins. The binding patterns facilitated the identification of amino acid residues of the compounds that bound to the protein pockets.

### Experimental verification

#### Drugs, reagents and antibodies

Artemisinin (#361593) powder was produced by Sigma-Aldrich (Shanghai) Trading Co.Ltd.. Its purity was more than 98% according to the data sheet from the manufacturer. The drugs were prepared with 5% sodium bicarbonate and were stored at 4 °C. Pilocarpine (#133246) was purchased from MedChemExpress. Hematoxylin Eosin staining (HE, #BA4025) was purchased from Gibco. FITC anti-mouse CD4 antibody (CD4 FITC, #100510), APC anti-mouse CD25 antibody (CD25 APC, #101910), and PerCP anti-mouse CD3ε antibody (CD3 PercP, #100326) were purchased from Biolegend. FOXP3 monoclonal antibody (FOXP3 PE, #12–5773-82), IL-17A monoclonal antibody (IL-17 PE-CY, #25–7177-82), and Foxp3/Transcription factor staining buffer set (#00–5523-00) were purchased from Invitrogen.

#### Animals and drug intervention

Nine-week-old female NOD/Ltj mice were purchased from Huafukang (Beijing) Biotechnology Co.Ltd., and female BALB/c mice in same age were selected from SPF (Beijing) Biotechnology Co.Ltd. as the normal group. Specific pathogen free NOD/Ltj mice are considered to be spontaneous SS models [23]. The mice were housed under a temperature of 23 ± 3 °C and a humidity of 50 ± 10% in the Experimental Animal Center of Beijing University of Chinese Medicine. All animal experiment procedures were approved by the Animal Care and Welfare Committee of Beijing University of Chinese Medicine (No. BUCM-4-2,021,090,307-3158). After 3 days of adaptive feeding, mice in the artemisinin group (*n* = 6) were intragastrically injected artemisinin (50 mg/kg) 0.20 mL daily. Mice in the HCQ group (*n* = 6) were administered HCQ (1.30 g/kg) 0.20 mL daily, while mice in the model group (*n* = 6) and normal group (*n* = 6) were treated daily with normal saline 0.20 mL at the same time. It was administered once daily for 8 weeks. Animals were divided into the following four groups each containing six animals (Fig. S1).

#### Histological analysis of salivary glands

In accordance with the principle of ethical respect for animal life in laboratory animals, animals will be disposed of in the least painful way. Eight weeks after treatment, the mice were sacrificed by inhalation of CO_2_ (compressed CO_2_, gas cylinder), and the spleen, submandibular gland, and peripheral blood were extracted. Three of the submandibular gland tissues were fixed and embedded in paraffin for HE staining. Chisholm-Mason method was used to evaluate the degree of lymphocytic infiltration. The pathological degree of lymphocytic infiltration in the submandibular gland of each group of mice was quantified (1 infiltrating foci means at least 50 lymphocytic infiltrations per 4 mm^2^), with 0 points for no or minimal lymphocytic infiltrations, 1 point for a few scattered lymphocytic infiltrations, 2 points for moderate lymphocytic infiltrations (no foci) with mild parenchymal damage. A score of 3 was given for < 1 lymphocytic infiltrate foci per 4 mm^2^ (4 low magnification views), and a score of 4 for > 1 lymphocytic infiltrate foci per 4 mm^2^. If the score is 2 or more, the disease is considered to have developed. Image Pro Plus (version 6.0) software was used to calculate the percentage of area infiltrated by inflammatory cells in each group of mice in the submandibular gland as quantitative staining results.

#### Saliva flow analysis

Three mice from each group were intraperitoneally injected with 1% sodium pentobarbital (50 mg/kg). After anesthetization, the mice were injected with pilocarpine (5 mg/kg). Saliva was collected from pilocarpine-injected mice with a dry cotton ball for 5 min and then removed and weighed for wet mass. The tissues of these three mice were not pathologized to prevent any effect on the histological results.

#### Flow cytometry

Spleens were prepared as single cell suspensions after sufficient grinding for flow cytometric analysis to detect CD4+ T cell subsets. The cell suspension was incubated with CD3 PercP, CD4 FITC and CD25 APC for 15 min. After fixation and permeabilization, FOXP3 PE and IL-17 PE-CY were added into the sample and incubated for 30 min and then measured by flow cytometry.

### Statistical analysis

Statistical analysis was performed using SPSS (Version 20.0) statistical package. Student’s t-test was used to detect the statistically significant differences between the two groups; ANOVA and the Dunnett’s test were used for comparisons of multiple groups. Data were presented as mean ± standard deviation. *P* < 0.05 was considered statistically significant.

## Results

### Related targets of artemisinin and pSS

**Fig. 1:**
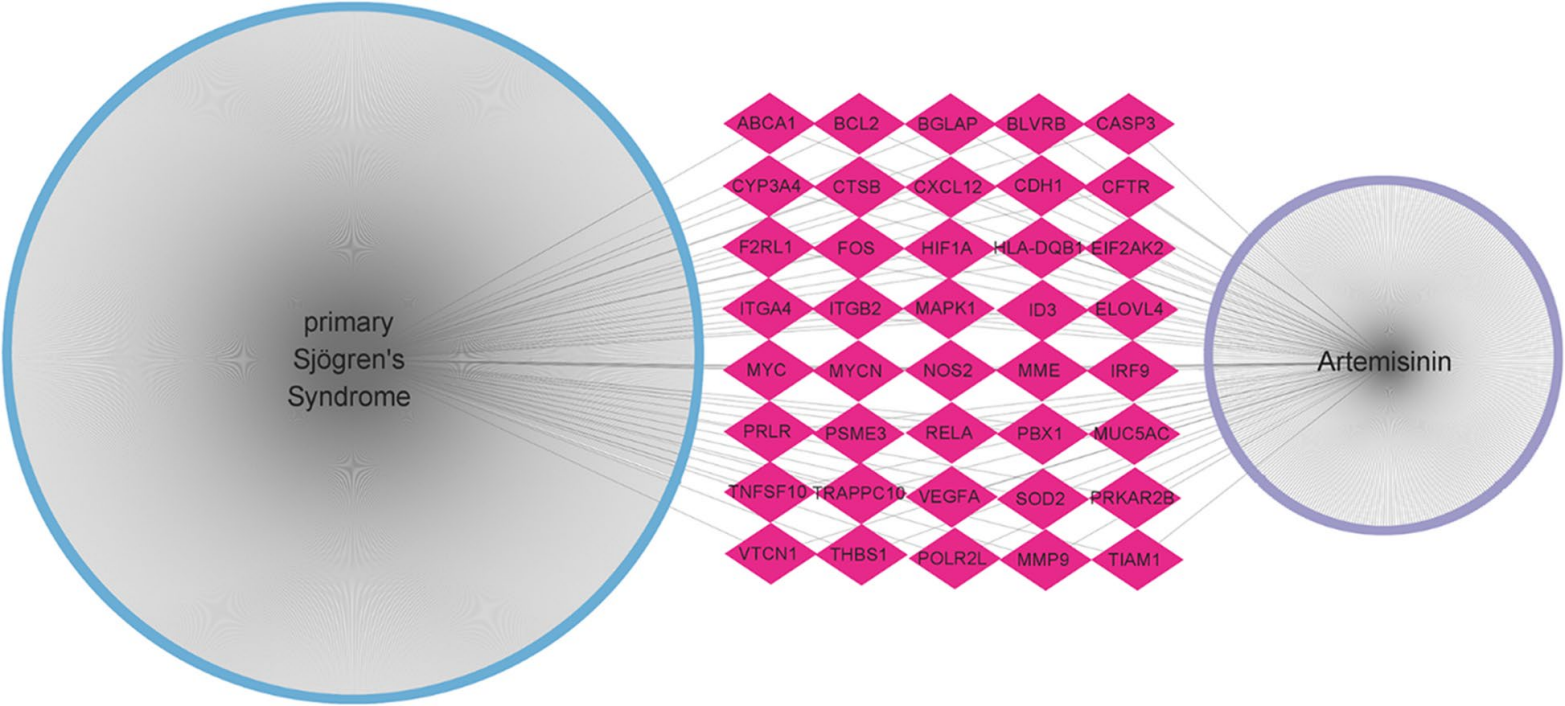
Artemisinin-pSS network

By searching the databases, 15, 409, and 8 artemisinin targets were obtained from the TCMSP, HERB, and CHEMBEL databases, respectively. After deleting duplicates, a total of 412 potential therapeutic targets were obtained. The targets of pSS were obtained from the OMIM, GeneCards, and DisGeNET databases, and a total of 1495 disease-related targets were included after screening. As shown in Fig. 1, among the obtained artemisinin-associated targets and pSS-associated targets, there were 40 intersection targets, suggesting that artemisinin may be involved in the treatment of pSS through these 40 targets.

The PPI network was constructed to predict the functional linkage of intersection targets. Each node represents a protein, and the content of the node is its 3D structure. The edges between the nodes denote protein-protein associations; line color indicates the type of interaction evidence; line thickness indicates the strength of data support. Fig. S2 showed that MMP9 had the highest confidence of interaction score, indicating that it made a great contribution to the PPI network. During the disease process, the targets are participating in the inflammatory signaling pathways (MMP9, MAPK1, CXCL2), apoptosis (CASP3, FOS, BCL2), cell autophagy (HIF1α), transcription regulation (RELA), and synovial neovascularization (VEGFA), which would be further validated by KEGG analysis.

### Enrichment analysis of the intersection targets

**Fig. 2:**
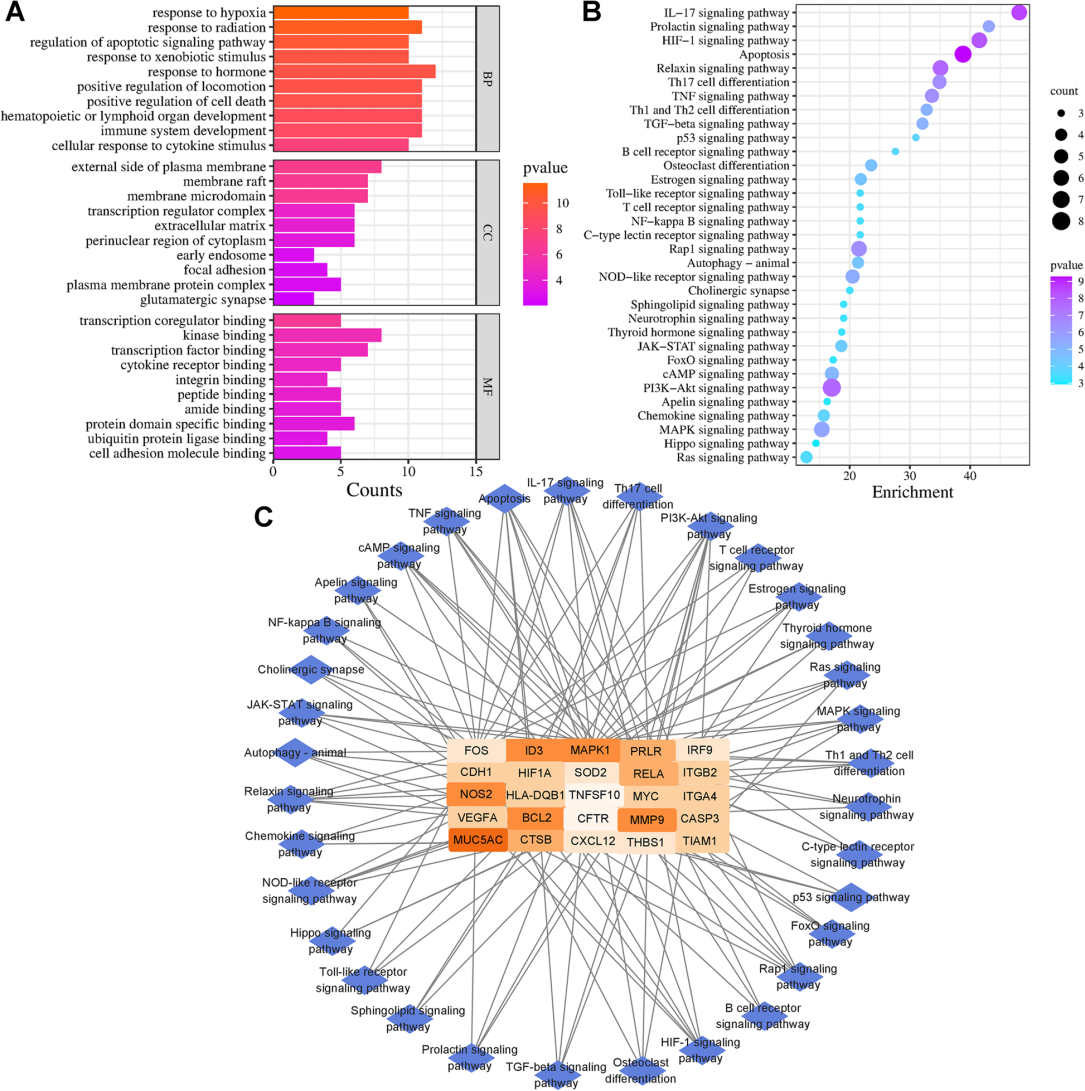
Network pharmacology predication of artemisinin against pSS. **A**, **B** The potential biological mechanism and pathway of artemisinin in the treatment of pSS. **C** Network of pSS-related pathways identified from KEGG enrichment analysis and their corresponding targets

GO analysis revealed that biological processes at the intersection of artemisinin-related and pSS-related genes were enriched in response to hypoxia, cytokine stimulation, and hormones. The cellular components in which the therapeutic targets of the targets were primarily located include membrane raft, membrane microdomain, extracellular matrix, and perinuclear regions of the cytoplasm. The molecular function of the targets was focused on the binding of transcription factor, cytokine receptor, and other small molecule substances. A biological function barplot diagram was drawn based on GO results (Fig. 2A).

The 40 intersection genes of pSS and artemisinin were used for KEGG enrichment analysis and then the top 33 pathways identified. A pathway dotplot diagram was drawn based on KEGG results (Fig. 2B). Among the top 33 pathways, IL-17 signaling pathway, HIF-1 signaling pathway, apoptosis signaling pathway, Th17 cell differentiation, PI3K-Akt signaling pathway, MAPK signaling pathway, Rap1 signaling pathway, TNF signaling pathway and TLR signaling pathway were the key pathways of artemisinin in the treatment of pSS. The 25 core proteins of key pathways predicted by KEGG analysis were subjected to molecular docking to further analyze the interaction of artemisinin with target proteins, while the IL-17 signaling pathway was applied to the experimental validation. A network of pSS-related targets and their corresponding pathways is shown in Fig. 2C; the therapeutic pathway of artemisinin for pSS via IL-17 signaling pathway was shown in Fig. S3.

### Interactions of artemisinin with different target proteins

**Fig. 3:**
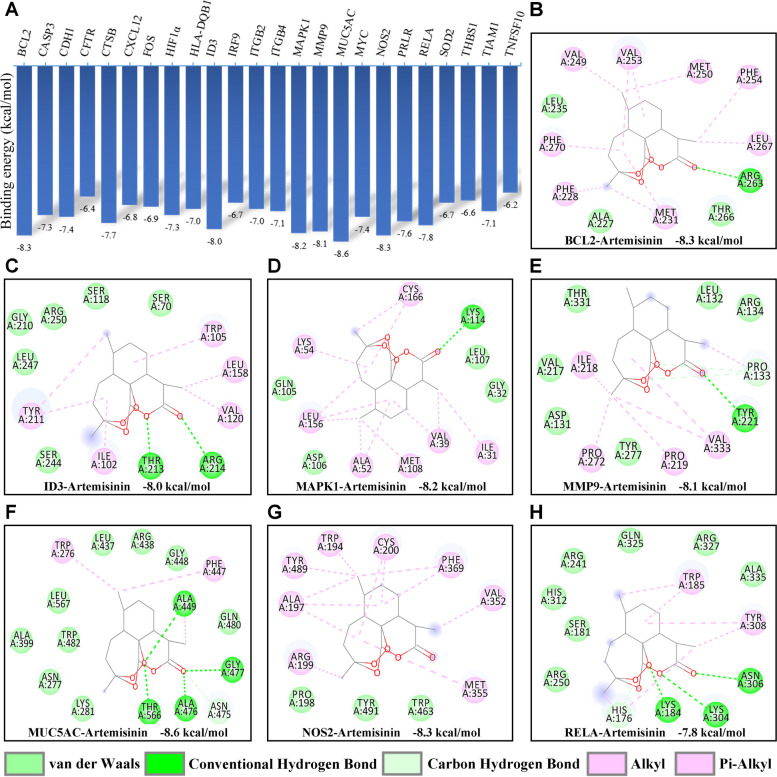
Molecular docking simulation of artemisinin with kernel targets. **A** The results of the binding energy between artemisinin and the protein. **B**–**H** The docking pattern of artemisinin with the affinity top 7 kernel protein respectively

In this experiment, artemisinin was molecularly docked to 25 target proteins. The molecular docking results showed that the compounds bound well to the target proteins with a good match (binding energy less than − 6 kcal/mol). The details of the binding energy were shown in Fig. 3A. As we can learn from the docking pattern of molecular binding (Fig. 3B-H), artemisinin contains multiple carbon rings and is able to form strong hydrophobic interactions with amino acids VAL-249, VAL-253, MET-250, PHE-254, LEU-267, MET-231, PHE-228, PHE-270, reflecting its strong hydrophobicity. Artemisinin also has strong hydrogen bonding interactions and hydrophobic interactions with the active sites of the other six protein targets. These interactions can effectively anchor small molecules in the protein sites, and thus be strongly associated with the protein targets. The results of molecular docking showed that the artemisinin molecule had higher binding energy by combining with the key nodes in IL-17 signaling pathway.

### Saliva flow rate and pathological changes of glands

**Fig. 4:**
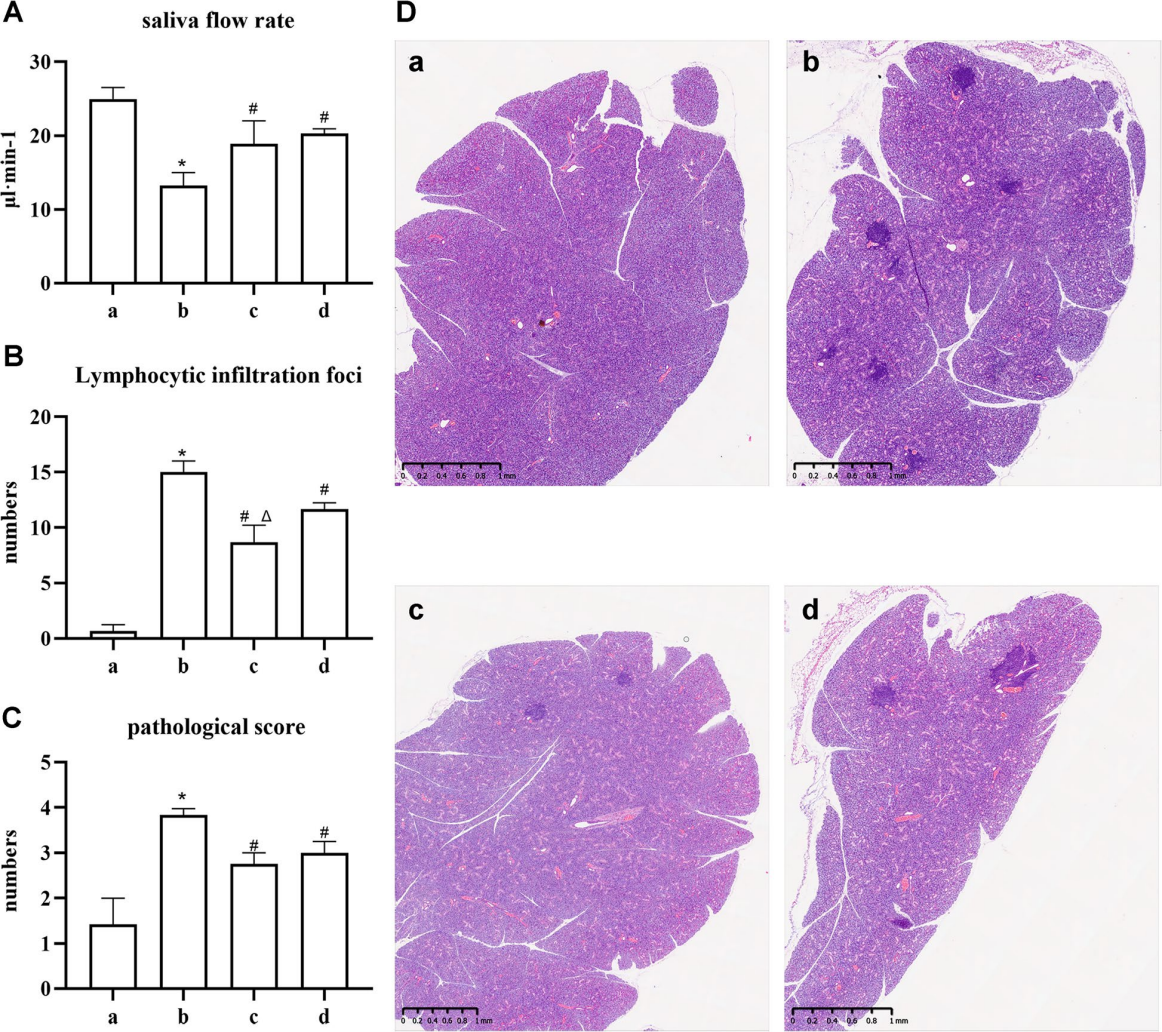
Artemisinin treatment reduced infammatory infltration and improved saliva fow rate in NOD/Ltj mice. **A** The saliva flow rate of each group. **B** The number of lymphocytic infiltration foci in each group. **C** The pathological score of each group. **D** HE staining of submandibular gland of mice in each group. a: normal group, b: NOD/Ltj model group, c: artemisinin group, d: HCQ group (*P < 0.05 compared with the normal group; #*P* < 0.05 compared with the model group; Δ*P* < 0.05 compared with the HCQ group)

After 8 weeks of treatment, the saliva flow rate of mice in the normal group remained basically stable (*P >* 0.05), while the saliva flow rate of mice in the model group was lower than before (*P* < 0.05). Compared with the normal group, the saliva flow rate of mice in the other three groups was significantly decreased (*P* < 0.05, Table 1). However, mice in the artemisinin and HCQ groups exhibited higher saliva flow rates than those in the model group (*P* < 0.05), indicating that a certain concentration of artemisinin could effectively restore the glandular secretion function, similar to the effect of HCQ treatment (Fig. 4A).

**Table 1:** Effect of artemisinin on salivary flow rate in mice

GROUP	Salivary flow rate at 0 week(μl min-1)	Salivary flow rate at 8 week(μl min-1)	*P*
normal group	28.21 ± 2.35	24.97 ± 1.54	0.282
model group	28.98 ± 1.50	13.25 ± 1.72^*^	0.012
artemisinin group	28.94 ± 2.10	18.88 ± 3.10^*#^	0.060
HCQ group	28.96 ± 1.70	20.31 ± 0.62 ^*#^	0.017

*P < 0.05 compared with the normal group; #*P* < 0.05 compared with the model group

The number of lymphocytic infiltration foci and the pathological score of the submandibular gland were performed to evaluate the degree of glandular damage (Fig. 4B-D). The model, artemisinin, and HCQ groups owned more lymphocytic infiltration foci and higher pathological scores than the normal group (*P* < 0.05). Meanwhile, less lymphocytic infiltration and lower pathological score were observed in the artemisinin group compared with the model group (*P* < 0.05). Among them, the number of lymphocyte infiltration foci was reduced in the artemisinin group compared with the HCQ group (*P* < 0.05), suggesting that artemisinin more effectively attenuated lymphocyte infiltration and improved the level of glandular damage of SS-like NOD/Ltj model mice.

### The proportion of Tregs and IL-17 in lymphocytes

**Fig. 5:**
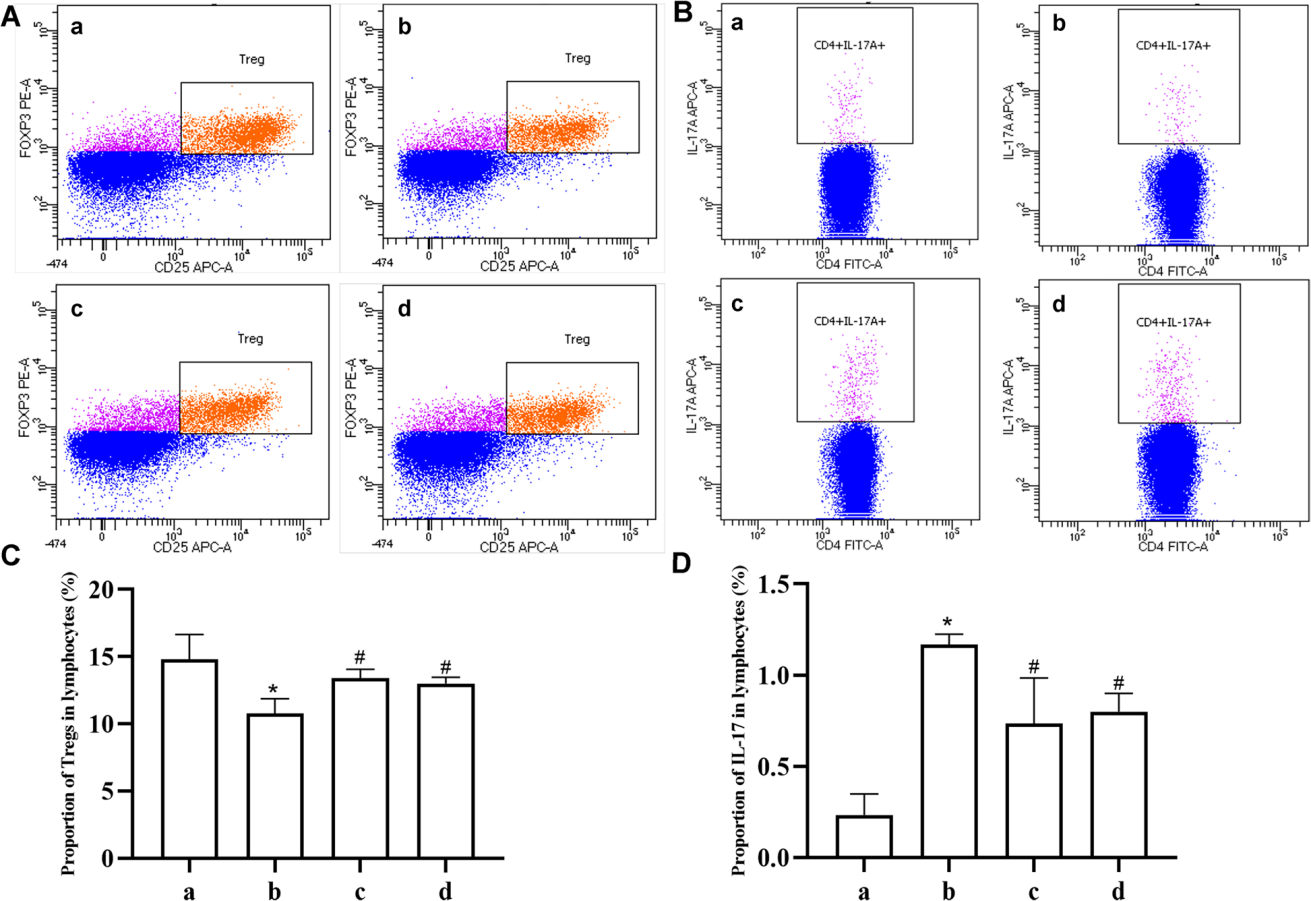
Artemisinin reduced Th17 cells and expanded Tregs in NOD/Ltj mice. **A** and **B** Tregs and IL-17 in lymphocytes measured by flow cytometry. **C** The proportion of Tregs. **D** The proportion of IL-17. a: normal group, b: NOD/Ltj model group, c: artemisinin group, d: HCQ group (*P < 0.05 compared with the normal group; #*P* < 0.05 compared with the model group)

The results of flow cytometry suggested that the proportion of Tregs in lymphocytes from model rats exhibited a significant decrease compared with the normal rats (*P* < 0.05) (Fig. 5A). The intervention of artemisinin and HCQ can increase the proportion of Tregs (Fig. 5C). The contents of IL-17 secreted by lymphocytes in model group was significantly higher than that in the normal group (*P* < 0.05) (Fig. 5B). Artemisinin and HCQ reversed this abnormal increase to some extent, with the former showing a more pronounced effect (Fig. 5D).

## Discussion

In this study, we predicted key targets and pathways in the treatment of pSS with artemisinin via network pharmacology and molecular docking. Experimental verification validated the predicted mechanism of the treatment of pSS with artemisinin in vivo.

Through network pharmacological analysis, 40 target genes were identified as potential targets for artemisinin treatment of pSS. GO enrichment analysis identified potential targets of artemisinin action, which are involved in many biological processes related to pSS pathogenesis, such as cellular hypoxia response, cytokine activation and signaling. The core target, MMP9, has the function of degrading and remodeling the extracellular matrix, which may inhibit the repair and regeneration of glandular epithelial cells by participating in the degradation of the basal layer components of the basement membrane cells, and ultimately activate apoptosis, which may result in the damage of the exocrine gland cells to a certain extent [24]. It was found that MMP9 expression was abnormally elevated in the salivary glands of SS patients, suggesting that artemisinin may ameliorate the inflammatory response and glandular damage in patients by inhibiting the abnormal secretion of MMP9 [25, 26]. KEGG enrichment analysis showed that artemisinin may improve pSS mainly by inhibiting inflammatory signaling pathways and immune-inflammatory responses. Among them, disturbances in the signaling cascades of the pathways IL-17, TGF-β, TNF, and TLR lead to the induction and development of pSS [27, 28]. Finally, the results of molecular docking between the drug and the disease target further verified that artemisinin possesses good molecular binding activity with the relevant core targets, indicating that artemisinin molecules may affect cellular immune-inflammatory responses through effective binding with core targets such as MMP9, MAPK1, and other core targets, which may play a role in the treatment of pSS.

The results of the animal experiment showed that the SS-like NOD/Ltj model mice had a reduced saliva flow rate, an increased percentage of lymphocytic infiltration foci and significant pathological changes. After artemisinin intervention, the degree of saliva flow rate elevated to a certain extent, and the pathological findings of glands were decreased, suggesting that artemisinin can suppress further structural damage as well as functional abnormalities in the submandibular gland of SS-like NOD/Ltj model. HCQ, a drug originally used as an antimalarial, is now used specifically for rheumatic autoimmune diseases, such as SLE, RA, and pSS, thanks to its low cost, safety and efficacy [29]. Recent studies have explained the possible mechanisms. At the cellular level, HCQ leads to reduced production and release of pro-inflammatory cytokines through multifaceted effects on different immune cells, such as inhibition of antigen presentation, B- and T-cell activation, and NOX signaling [30–33]. In addition, it rebalances the Treg/Th17 cell ratio [34–36]. However, prolonged administration of HCQ may still cause gastrointestinal disorders, skin discoloration or rashes, elevated muscle enzymes, and even more severely, retinopathy, neuromuscular, and cardiac toxicity [37]. Our experiments show that artemisinin has almost equal efficacy to HCQ, suggesting a potential clinical utility of artemisinin in the treatment of pSS.

Immune dysregulation of T lymphocytes and their mediated abnormalities in B lymphocyte immunity are important mechanisms in the pathogenesis of pSS [38]. Th cells play an important role in stimulating abnormal activation of B cells to produce autoantibodies [39]. Previous studies have shown that the majority of lymphocytes infiltrating the glands of pSS patients are CD4^+^ T cell subsets, including Th1/Th2 cells and Th17/Treg cells in a state of immune imbalance [40, 41]. As a new subpopulation of CD4^+^ T cells, Th17 cells play a crucial role in autoimmune diseases and chronic inflammatory responses [42]. IL-17 is the primary inflammatory cytokine secreted by Th17, inducing the expression of various cytokines such as pro-inflammatory cytokines, chemokines and matrix metalloproteins via IL-17 signaling pathway [43]. The expression of IL-17 has been found to correlate with the severity of glandular inflammation in several studies [44]. Follow-up study has also shown that blocking the IL-17 signaling pathway can inhibit the pathways involved in Th cell-assisted B cells differentiation and maturation [45]. In in vivo experiments, IL-17 expression was significantly increased in lymphocytes, suggesting increased numbers and/or enhanced activity of Th17 cells, indicating aberrant differentiation of Th17 cells in NOD/Ltj mice. This change was reversed by artemisinin, suggesting that artemisinin may inhibit Th17 cell differentiation through the T cell receptor signaling pathway. Tregs play an important role in the maintenance of self-tolerance, and exert suppressive activity on autoreactive lymphocytes, mainly through the secretion of suppressive anti-inflammatory cytokines, such as IL-10 and transforming growth factor (TGF)-β [46]. Forkhead box protein P3 (Foxp3) is a Treg-specific marker [47]. The results suggested that the expression of Tregs in the lymphocytes from NOD/Ltj mice showed a marked decrease. The lymphocytes of artemisinin and HCQ groups exhibited a significant increase of Tregs, indicating the decreased in immune response intensity. These results suggested that the inflammation was inhibited to a certain degree post-treatment.

Upon antigen stimulation, naive CD4^+^ T cells undergo activation, expansion and differentiation into different effector sub-populations [48]. TGF-β and IL-6 are key cytokines for the induction of the Th17 phenotype, while TGF-β and IL-2 are molecules required for the differentiation of Treg cells [49]. TGF-β signaling pathway is shared by Th17 and Treg cells. In the presence of both IL-6 and TGF-β, naive CD4^+^ T cells can activate signal transducer and activator of transcription 3 (STAT3) through phosphorylation, and induce the expression of various Th17 cell-specific genes such as retinoid-related orphan nuclear receptor γt (RORγt) to promote Th17 cell differentiation [50]. STAT3 phosphorylation can also inhibit Treg cell differentiation, mainly by suppressing the expression of Foxp3 activated by TGF-β [51]. If phosphorylated, the TGF-β signaling pathway activates SMAD Family Member 2 (Smad2) and Smad3, the transcription factors Sma and Mad related proteins, which bind to the Foxp3 motif [52]. At the same time, IL-2 signaling can promote Foxp3 expression by phosphorylating signal transducer and activator of transcription 5 (STAT5), preventing the differentiation of naive CD4^+^ T cells into Th17 cells [53, 54]. In conclusion, the physiological functions of Th17 and Treg cells are antagonistic, and the differentiation of these two cells is regulated by the interaction of various cytokines. When antigen-driven inflammation persists, interconversion between Th17 and Treg may occur [55], since the cytokine network appears to favor the differentiation of Th17 cells while minimizing immunosuppressive Tregs [56]. This imbalance between pro- and anti-inflammatory forces persists in autoimmune pathologies such as pSS.

In our study, network pharmacology suggested that IL-17 signaling pathway is a core pathway in the treatment of pSS with artemisinin. Accumulating evidence indicates that the pro-inflammatory cytokine IL-17 is involved in the occurrence and progression of pSS [57–59]. IL-17 disrupts the integrity of the barrier formed by the tight junctions of glandular tissue cells through the NF-κB signaling pathway, thus leading to impaired secretory function of the SS salivary glands [60]. It has been demonstrated that inhibition of IL-17 in the early stages of SS disease can prevent further progression of SS, especially by reducing lymphocyte infiltration into the salivary gland and maintaining normal salivary volume; similarly, blocking IL-17 in the late stages of the disease can save the function of the gland by reducing lymphocyte infiltration into the salivary gland [61]. Therefore, active blockade of IL-17 in the early stages of the disease has an important role in improving and restoring salivary gland function. Artemisinin has a potential role in blocking the IL-17 signaling pathway.

## Conclusion

Taken together, we can conclude from our results that artemisinin can can restored salivary gland secretory function and improve the level of glandular damage of SS mice, and its mechanism may be closely related to inhibiting the differentiation of Th17 cells and the secretion of inflammatory factors (IL-17) by lymphocytes and modulating the balance of Tregs and Th17 cells. Further exploration of the optimal effective dose of artemisinin for the treatment of pSS, and the relevant factors that can guide dose adjustment is needed in the future, which is of great significance for the clinical treatment of artemisinin applied to pSS.

### Supplementary Information

**Additional file 1.**

**Additional file 2.**

**Additional file 3.**

**Additional file 4.**

## Data Availability

The data used to support the findings of this study are included within the manuscript and Supplementary Materials.
