# Supplementary figures and images for: Network pharmacology-based strategy to investigate the mechanisms of artemisinin in treating primary Sjögren’s syndrome
Source: BMC Immunol. 2024 Feb 12;25:16. doi: 10.1186/s12865-024-00605-3 (PMC10860289; doi:10.1186/s12865-024-00605-3)

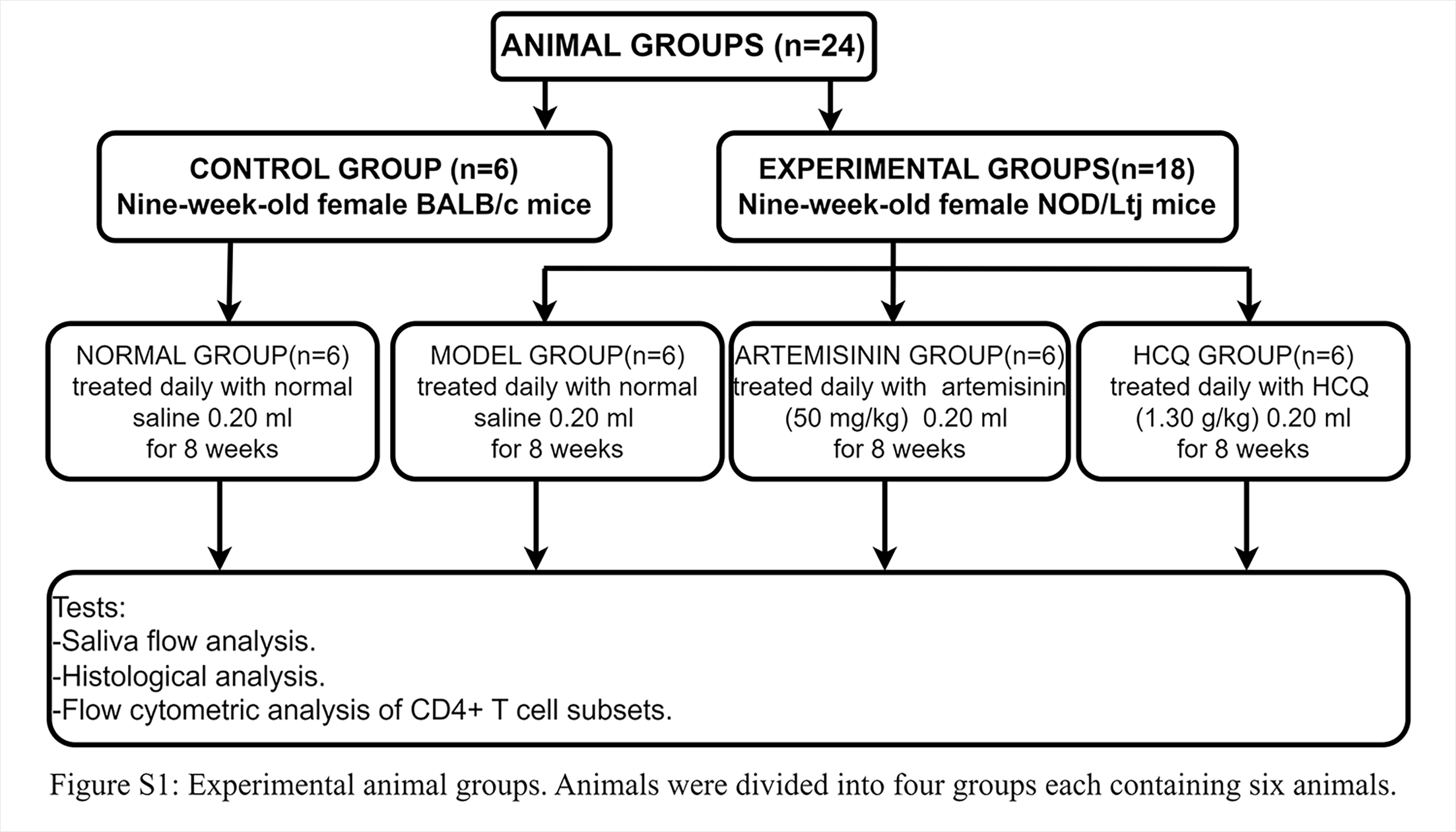

Supplement: Supplementary file 1 — Additional file 1. [file 12865_2024_605_MOESM1_ESM.tif]

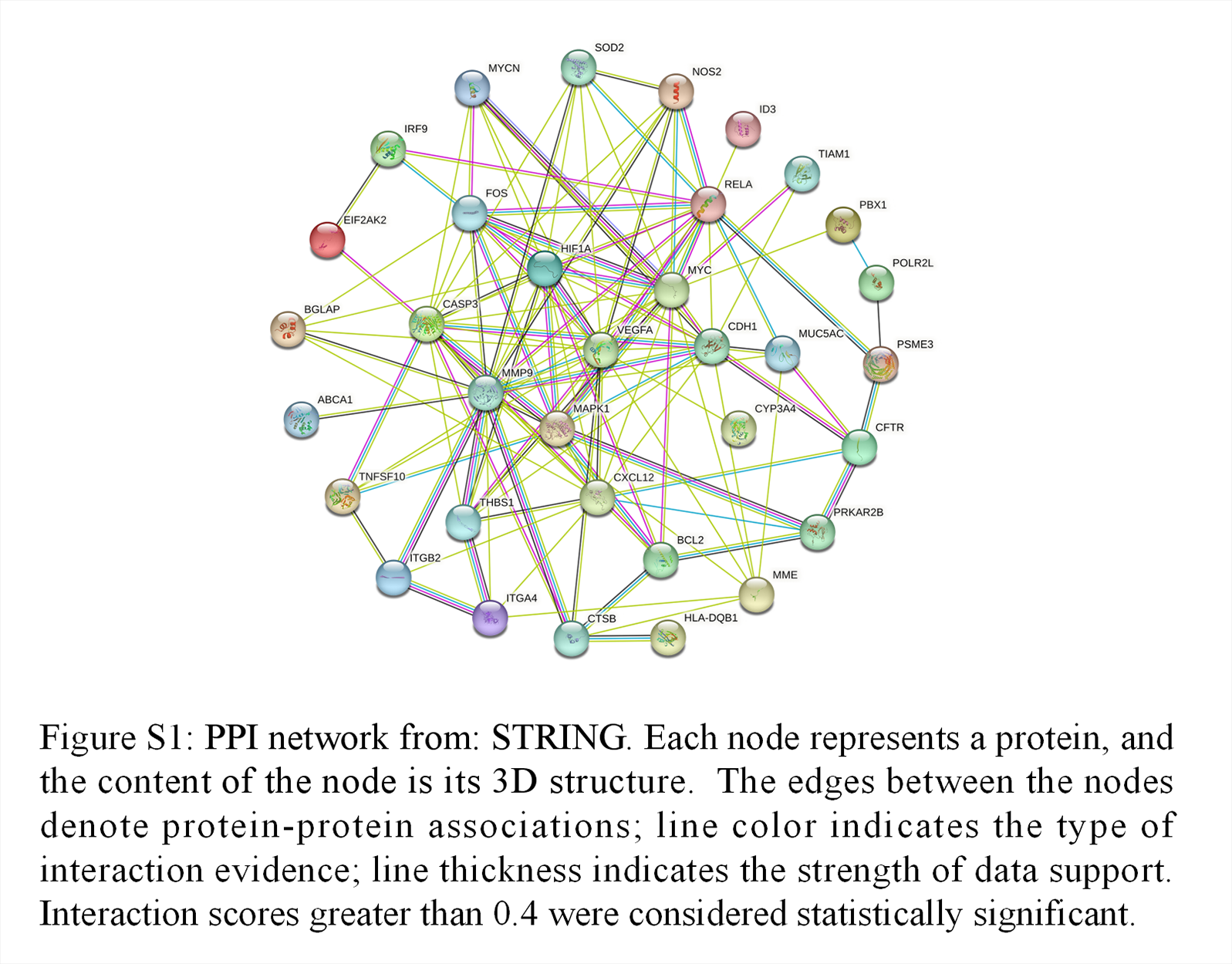

Supplement: Supplementary file 2 — Additional file 2. [file 12865_2024_605_MOESM2_ESM.tif]

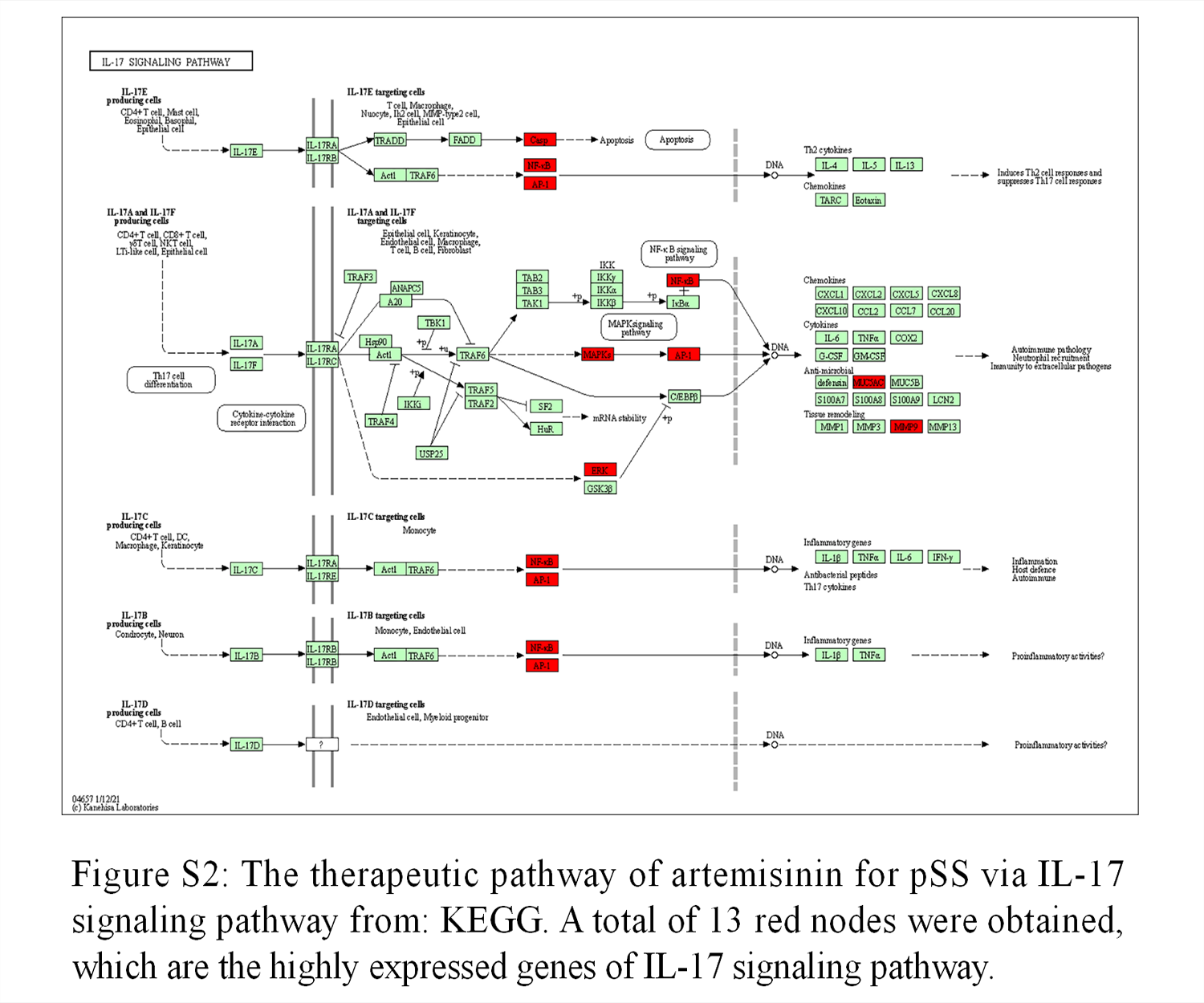

Supplement: Supplementary file 3 — Additional file 3. [file 12865_2024_605_MOESM3_ESM.tif]
